# A review of the risk and precipitating factors for spontaneous coronary artery dissection

**DOI:** 10.3389/fcvm.2023.1273301

**Published:** 2023-12-19

**Authors:** Dragana Stanojevic, Svetlana Apostolovic, Tomislav Kostic, Vladimir Mitov, Dusanka Kutlesic-Kurtovic, Mila Kovacevic, Jelena Stanojevic, Stefan Milutinovic, Branko Beleslin

**Affiliations:** ^1^ Clinic for Cardiology, University Clinical Center Nis, Nis, Serbia; ^2^ Internal Medicine Department, Medical Faculty University of Nis, Nis, Serbia; ^3^ Department for Cardiovascular Diseases, Health Center Zajecar, Zajecar, Serbia; ^4^ Clinic for Cardiology, Institute for Cardiovascular Diseases Vojvodina, Novi Sad, Serbia; ^5^ Internal Medicine Department, Medical Faculty University of Novi Sad, Novi Sad, Serbia; ^6^ Internal Medicine Residency Program, Florida State University College of Medicine, Cape Coral, FL, United States; ^7^ Clinic for Cardiology, University Clinical Centre Serbia, Belgrade, Serbia; ^8^ Internal Medicine Department, Medical Faculty Belgrade, Belgrade, Serbia

**Keywords:** spontaneous coronary artery dissection, non-traditional risk factors, fibromuscular dysplasia, pregnancy, emotional stress

## Abstract

**Introduction:**

Spontaneous coronary artery dissection (SCAD) accounts for 1%–4% of cases of acute coronary syndrome (ACS). SCAD is caused by separation occurring within or between any of the three tunics of the coronary artery wall. This leads to intramural hematoma and/or formation of false lumen in the artery, which leads to ischemic changes or infarction of the myocardium. The incidence of SCAD is higher in women than in men, with a ratio of approximately 9:1. It is estimated that SCAD is responsible for 35% of ACS cases in women under the age of 60. The high frequency is particularly observed during pregnancy and in the peripartum period (first week). Traditional risk factors are rare in patients with SCAD, except for hypertension. Patients diagnosed with SCAD have different combinations of risk factors compared with patients who have atherosclerotic changes in their coronary arteries. We presented the most common so-called “non-traditional” risk factors associated with SCAD patients.

**Risk factors and precipitating disorders which are associated with SCAD:**

In the literature, there are few diseases frequently associated with SCAD, and they are identified as predisposing factors. The predominant cause is fibromuscular dysplasia, followed by inherited connective tissue disorders, systemic inflammatory diseases, pregnancy, use of sex hormones or steroids, use of cocaine or amphetamines, thyroid disorders, migraine, and tinnitus. In recent years, the genetic predisposition for SCAD is also recognized as a predisposing factor. The precipitating factors are also different in women (emotional stress) compared with those in men (physical stress). Women experiencing SCAD frequently describe symptoms of anxiety and depression. These conditions could increase shear stress on the arterial wall and dissection of the coronary artery wall. Despite the advancement of SCAD, we can find significant differences in the clinical presentation between women and men.

**Conclusion:**

When evaluating patients with chest pain or other ACS symptoms who have a low cardiovascular risk, particularly female patients, it is important to consider the possibility of ACS due to SCAD, particularly in conditions often associated with SCAD. This will increase the recognition of SCAD and the timely treatment of affected patients.

## 1. Introduction

Approximately 1%–4% of patients diagnosed with acute coronary syndrome (ACS) are predicted to have spontaneous coronary artery dissection (SCAD), which challenges the perception that SCAD is a rare disease. SCAD is characterized by separation occurring within or between any of the three tunics of the coronary artery wall which is not caused by trauma. Thus, the formation of an intramural hematoma and/or false lumen occurs, resulting in myocardial infarction (MI) in the form of either STEMI (up to 37%) or NSTEMI (53%–59%). In addition, it leads to sudden cardiac death in 3.6%–10% of cases (1, 2).

SCAD is not associated with coronary atherosclerosis and lipid accumulation. The development of SCAD is explained with two theories. The first theory explains that the formation of intramural hematoma is caused by the tearing of the innermost tunica of the coronary artery, while the second theory is focused on the outer layer and the rupture of its blood vessels, resulting in similar consequences (3, 4).

After careful examination of coronary angiograms, the frequency of SCAD was reported to be between 23% and 36% in women under the age of 60. This high prevalence indicates that this condition is frequently misdiagnosed (2, 5). During pregnancy or the peripartum period, ACS is caused by SCAD in up to 43% of cases (1, 6, 7).

SCAD predominantly affects the left anterior descending artery, in approximately 50% of cases, with the left circumflex and right coronary arteries being affected subsequently. Over 90% of patients experience dissection in the mid and distal segments of epicardial arteries, while 13% of cases involve the left main artery, and approximately 5%–10% of cases affect two or more coronary arteries (4).

In most prospective registries, SCAD is most frequently diagnosed in Caucasians and predominantly in women compared with men (9:1). In certain observational cohorts, there is a deviation from the typical trend, with men being more frequently diagnosed with SCAD than previously reported. In one study with more than 30,000 patients with ACS, even 1.2% of patients were found to have a diagnosis of SCAD, with males accounting for 35.7% of these cases (1, 8). Published literature indicates that men with SCAD were significantly younger than women (approximately 48 vs. 52 years).

**Figure 1:**
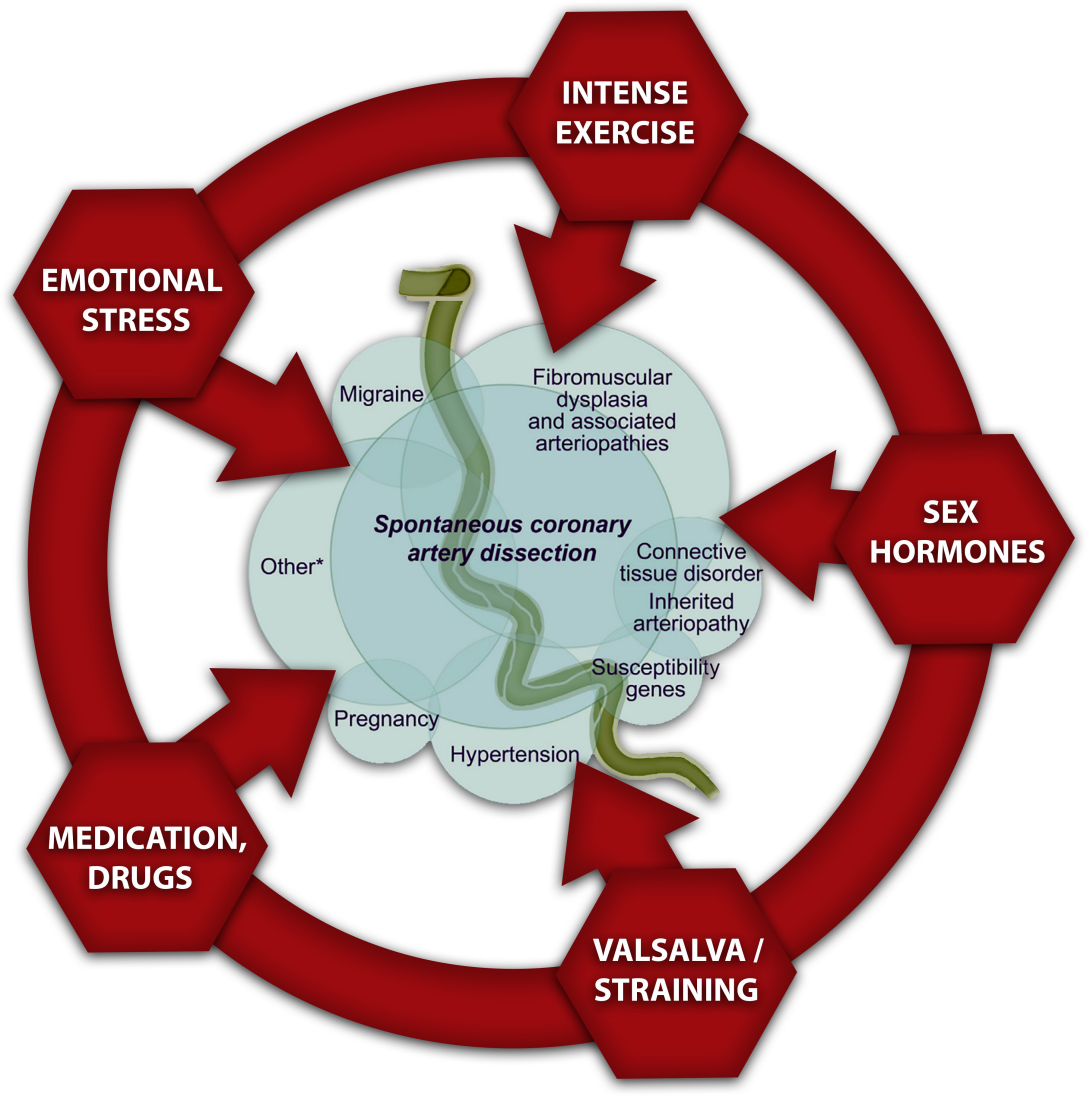
Risk and precipitating factors for SCAD.

The pathophysiology of SCAD is remains uncertain; however, it is possibly that a combination of risk factors with predisposing conditions and precipitating factors facilitate its development. A few risk factors such as female sex, pregnancy, and fibromuscular dysplasia (FMD) are strongly linked with SCAD in multiple studies. The link between SCAD and other reported associated conditions or risk factors has not been established, as previously mentioned (Figure 1) (10).

SCAD is often referred to as disease in patients without the traditional risk factors for coronary artery disease (11). However, traditional risk factors such as hypertension, hyperlipidemia, smoking, and obesity are present in those patients. Further, hypertension is found in almost similar percentage of patients with SCAD compared with age-matched population. The presence of traditional risk factors does not exclude the diagnosis of SCAD in young patients with ACS (12).

SCAD-related risk factors such as migraine and FMD should be determined with the goal of precise and prompt diagnosis, adequate therapy, and prognosis in this potentially fatal and recurrent disease. In addition, it is crucial to recognize the preceding precipitants that trigger SCAD, such as physical or emotional distress, which are more prevalent in SCAD than in ACS caused by atherosclerosis, for comprehensive management. Recently published studies have shown that emotional stressors are more prevalent in SCAD than in MI caused by atherosclerosis (56% vs. 17%), and similarly, physical exertion are more prevalent in SCAD than in MI caused by atherosclerosis (24% vs. 14%) (3).

We present the review of the prevalent non-traditional risk factors associated with SCAD, which are important for diagnosis, treatment, individual rehabilitation programs, and ultimately, patient prognosis.

### 1.1. Female sex and pregnancy

The highest percentage of SCAD patients (approximately 90%) are perimenopausal women. The predominance of females among SCAD patients and its high prevalence in pregnancy suggest that sex hormones play a significant role in the pathophysiology of this condition. The precise mechanisms of this pathophysiology association are still undetermined. Female sex hormones have an impact on arterial connective tissue and microvasculature. The obtained results suggest that the risk factors for SCAD in pregnancy (P-SCAD) include having more than one pregnancy, using sex hormones for *in vitro* fertilization (IVF), and having pre-eclampsia (6, 10).

Most events are reported during the last trimester and during the first week of the postpartum period. SCAD may occur even in the late postpartum (6–12 months) period, and it is linked with breastfeeding (10). A comprehensive study conducted in Canada between 2008 and 2012 revealed a higher incidence of P-SCAD compared with the data from prior studies (1.8 cases per 100,000 pregnancies). In the same study, P-SCAD patients exhibited worse prognosis than SCAD females who were not pregnant (NP-SCAD) having more frequently STEMI (64%), cardiogenic shock (24%), cardiac arrest (14%), and death (4.5%). P-SCAD patients exhibited a higher incidence of proximal coronary artery dissection and a significantly decreased left ventricle ejection fraction (LVEF) of ≤35%. This data regarding the poor prognosis in P-SCAD compared with NP-SCAD patients was confirmed in another American study (10).

Patients with P-SCAD compared with NP-SCAD had a higher prevalence of black ethnicity, older, with a history of arterial hypertension and hyperlipidemia, more often had depression, migraines, and more frequently were treated for infertility (1).

The hormonal and hemodynamic changes that occur during pregnancy, along with the increased shear stress on the coronary arteries, may contribute in developing SCAD. Within the initial 6 weeks of pregnancy, hemodynamics starts to change, including an increase in blood volume and in red blood cell mass while systemic vascular resistance is decreasing, and consequently, the cardiac output is higher. These changes are expected, but they increase the oxygen demands of the myocardium and could lead to developing SCAD. As previously stated, the highest incidence of P-SCAD was observed in the first month after childbirth, with the peak of incidence observed within the first postpartum week. During this period, there is another rapid increase in cardiac output due to the fast return of blood from the contracted uterus. Progesterone and estrogen have the highest levels during this period, which could be important since the endothelium has the sex hormone receptors. Estrogen induces the release of nitric oxide, resulting in vasodilatation, but it also influences the function of matrix metalloproteinases, which degrades the components of the artery wall and therefore contributes to SCAD (2–6). SCAD was also reported during lactation when oxytocin is released, which affects vasculature. Importantly, one of the metabolic products of oxytocin degradation is cardiotoxic and is found to be responsible for the occurrence of peripartum cardiomyopathy. This could also be important for the pathophysiology of SCAD (2–6).

### 1.2. Exogenous hormones

The use of oral estrogen and progesterone were linked to an increased risk of developing SCAD in few reports (one single-center study with case reports) (13). Estrogen replacement therapy has known effects on coagulation, microcirculation, increased release of reactive oxygen species, and triggering arrhythmias, all of which may potentially be associated with SCAD (13). However, in a study conducted by Saw et al. (14) on a group of 168 females with SCAD, no association was found between the active use of oral combined hormone therapy and the incidence of SCAD.

There is limited data available regarding SCAD occurrence after *in vitro* fertilization. In the United States, 9% of patients with P-SCAD underwent IVF. Women with P-SCAD more frequently underwent IVF treatment compared with women in childbearing age in the US population (28% vs. 12%) (15, 16). In this process, patients are administered gonadotropin therapy together with selective estrogen modulators and aromatase inhibitors to stimulate the production of follicles that secrete high levels of estrogen and progesterone. As previously mentioned, these hormones could cause weakening and vascular wall rupture. Clomiphene is the most frequently used selective estrogen receptor modulator in IVF, and previously, it has been linked with different pathological conditions such as MI and potentially with SCAD (16).

The role of estrogen and progesterone levels or their fluctuations in SCAD occurrence is unclear. A limited number of studies have explored SCAD development during menstruation, hormone replacement therapy, and in women undergoing or having undergone IVF treatment (17).

One hypotheses explaining the gender difference in SCAD is the various responses to androgens in the cardiovascular system and other organs. The X chromosome contains genes responsible for the expression of androgen receptors. In females, the different response to androgens is the result of autosomal mosaicism. It is known that estrogens are synthetized from androgen precursors, catalyzed by aromatase. Furthermore, women frequently exhibit specific coronary anatomy characterized by tortuous coronary vessels and a predisposition to microvascular dysfunction as a consequence of increased metalloprotease activity induced by estrogens (13).

### 1.3. Systemic arteriopathies

Connective tissue disorders, such as Marfan syndrome, Ehlers–Danlos syndrome type 4, and Loeys–Dietz syndromes, as well as polycystic kidney disease, are found in <5% of patients with SCAD (18, 19). The diagnosis of these conditions is important as it can guide familial screening and careful follow-up of affected patients (19).

Patients with SCAD in whom arterial imaging for extra-coronary vascular abnormalities was performed frequently exhibited fibromuscular dysplasia (18). The DISCO study is the first European multicenter analysis of SCAD patients and one of the largest studies in the world. The prevalence of FMD in this large cohort of SCAD patients was found to be 45% (20).

Marfan syndrome is caused by mutations in the fibrillin-1 gene (*FBN1*), and, among other abnormalities, patients with this syndrome exhibit dilatation of the aortic sinus with an increased risk of type A aortic dissection. There is a limited number of case reports in the literature regarding SCAD in patients with Marfan syndrome, and these reports suggest that the association between these entities lies in the structural changes in the connective tissue of the artery wall, which predispose to vessel dissection (2, 21).

In vascular Ehlers–Danlos syndrome, mainly middle-sized muscular arteries are affected, namely, mutations in the alpha 1 gene (*COL3A1*) are the cause of this syndrome. Collagen type III synthesis is disrupted, and consequently, patients are at risk for vascular rupture. SCAD has been reported to be associated with this syndrome in a limited number of case reports (2, 22).

Loeys–Dietz syndrome is caused by mutations in genes encoding compounds involved in the TGF-beta signaling pathway. Patients with this syndrome have tortuous arteries and arterial aneurysms with a high risk of arterial dissection or rupture. The risk remains high even in arteries that have normal diameters (2, 23).

Autosomal dominant polycystic kidney disease (ADPKD) was associated with SCAD in a few case reports. Patients with ADPKD have mutations in the polycystic kidney disease-1 and 2 genes that encode the polycystin synthesis. Polycystin is important for the integrity of the vascular wall, and its absence leads to vascular wall rupture and hemorrhage (2, 13).

### 1.4. Fibromuscular dysplasia

Fibromuscular dysplasia is the most frequently associated with SCAD in the literature. However, the most frequent clinical presentation of this arteriopathy is renovascular hypertension. The association with SCAD was first reported in 2011 by Saw et al. This is a disease of medium-sized artery walls that is not caused by atherosclerosis or inflammation. The highest incidence is found in middle-aged women who do not have many traditional risk factors. FMD is characterized by changes in the arteries, such as stenosis and aneurysms, which make arteries susceptible to dissection. The renal, cervico-cephalic, and visceral arteries are commonly involved (2, 10).

Angiographically, FMD has two types: multifocal, which is predominant, and unifocal. Between 41% and 86% of SCAD patients experience changes in other vascular beds, with the majority of them exhibiting changes in two or more extra-coronary vascular beds (29%–70%). The possible clinical manifestations of cervico-cephalic FMD could be ischemic stroke or subarachnoid bleeding due to arterial dissection or intra-cerebral aneurysm (2). Canadian authors reported the presence of cerebral aneurysm in 7.1% of SCAD patients (24).

In the American registry for FMD, the frequency of SCAD was less than 3%. Furthermore, in one large study with more than 60,000 ACS patients, it has been shown that 0.16% of cases caused by SCAD were linked with FMD. Even SLE, a specific systemic inflammatory disease, had a higher association rate with SCAD (13, 25, 26).

*PHACTR1/EDN1* is a genetic locus on chromosome 6q24 which carries the risk for coronary artery disease and AMI (27). This gene is also associated with migraine and cervicocerebral artery dissection (CeAD) (28). The putative variant of the *PHACTR1* locus was found near a putative regulatory genetic region for *EDN1*, the *endothelin (ET)-1* gene. This putative PHACRT1 variant relates to the risk of FMD. Interestingly the rs9349379-A allele is linked with a higher risk for FMD, CeAD, and migraine, whereas the less common allele, rs9349379-G, is linked with an increased risk for atherosclerosis. Firstly, the determined genetic risk locus for SCAD is rs9349379. The link between SCAD and FMD was determined even on the genetic level. Gupta et al. found that the rs9349379-A allele is found more frequently in patients with lower levels of circulating ET-1 than in healthy population. Endothelin-1 is a potent vasoconstrictor, and its vascular effects could have an important role in the pathophysiology of SACD. Whether the ET-1 expression decreased in SCAD and FMD is still to be determined (7, 29, 30, 31).

### 1.5. Inflammatory conditions

In the literature, SCAD was closely linked with the systemic inflammatory conditions, and the highest incidence was reported in one Canadian series (in 8.9% of cases). However, the mechanistic link between those two pathological conditions is still unknown. Since 1982, SCAD has been associated with inflammatory processes. This connection was initially observed when arterial biopsies revealed a focal infiltration of the tunica adventitia with inflammatory cells, predominantly eosinophils (5). Hypothetically, they could release lytic enzymes, causing dissection (30, 31).

Common systemic inflammatory disorders associated with SCAD include systemic lupus erythematosus (SLE), rheumatoid arthritis, celiac disease, Crohn's disease, and ulcerative colitis (19). A sub-analysis of the Framingham offspring study reported that women with SLE have a 50-fold increased risk of MI compared with age-matched control. Having said that, only several case reports documenting the occurrence of SCAD in SLE patients were published. SCAD can be the first manifestation of SLE, and therefore, screening for this disease is prudent in these patients (2, 19). In the literature, we found different inflammatory diseases in patients with SCAD such as sarcoidosis and infective hepatitis. Polyarteritis nodosa with pathological changes in small- and medium-sized arteries has also been associated with SCAD (2, 32–34). In these case reports or case series, coronary vasculitis or systemic inflammation may explain the increased risk for SCAD. In the larger cohort studies, a rather small percentage of patients had inflammatory conditions or short-term increases in inflammatory markers. This systemic inflammatory disease could only be a coincidental finding (1). The authors did not find a connection between SCAD and vasculitis in systemic arteries in the conducted studies. Coronary arteritis is a rare pathological condition, and it is characterized by the stenosis or occlusion of the proximal parts of arteries accompanied by skip lesions or aneurysms. This finding is not characteristic of SCAD (31).

Recently, a case report of SCAD in COVID-19-infected patient was published. It is known that COVID-19 is inducing a marked inflammatory and immune response which can damage endothelial and smooth muscle cells in blood vessels. Theoretically, the inflammatory response induced by the infection could promote the fragility of coronary vessels and lead to SCAD (35).

Patients with autoimmune disorders have an increased risk for SCAD (1). The frequency of autoimmune diseases in SCAD patients is comparable with the frequency in the general population (approximately 9%) (36). A recent study using population-based data from the Rochester Epidemiology Project showed that patients with autoimmune diseases did not have an increased risk for SCAD (31).

### 1.6. Migraine

In SCAD cohort studies, the reported prevalence rate of migraine ranged from 37% to 46%, contrasting with a lifetime migraine prevalence rate of 24% in women from the Women's Ischemia Syndrome Evaluation (WISE) cohort in the United States. Female SCAD patients have an estimated 37% higher age-adjusted 1-year migraine prevalence rate, as reported (37). Migraine is characterized by headache, typically unilateral in location, accompanied by nausea/vomiting and sensitivity to loud sounds and lights (38). Studies showed that endothelial dysfunction in migraines could be involved in developing stroke and in the pathogenesis of cervical artery dissection. It is postulated that a similar association could be present in SCAD. In a study conducted by the Mayo Clinic research group found that migraines were highly prevalent among the female population with SCAD, as previously reported. The same researchers found that migraineurs with SCAD were younger with more prevalent arterial abnormalities. They more frequently complained of chest pain and depression. In a large US national cohort study with 66,360 SCAD patients, only 0.8% of patients had migraine (13).

### 1.7. Thyroid disorders

Patients with SCAD had a higher prevalence of hypothyroidism when compared with individuals with atherosclerosis who develop ACS. Among SCAD patients with hypothyroidism, there was a higher incidence among women, and they exhibited frequent dissection in the distal segments of coronary arteries as well as dissections in the corkscrew arteries. Therefore, they were more often treated conservatively. Thyroid hormones have a significant role in the heart and blood vessels. Hypothyroidism is accompanied by conduction disturbances, different types of arrhythmias, rapid atherogenesis, and coronary artery disease. The results from studies conducted on a small number of patients indicate that hypothyroidism may somehow be involved in SCAD pathophysiology. In those patients, even iatrogenic coronary artery dissection is more frequent (39). Also, Spanish authors found a high percentage of autoimmune thyroid dysfunction in the population with SCAD (40).

### 1.8 Inheritance and genetics

In ≤5% of SCAD cases, we found hereditary arteriopathies and connective tissue disorders (18). However, Goel et al. (41) published a case series with five pairs of relatives with angiographically confirmed SCAD.

Several studies investigating the susceptibility genes for SCAD were published in 2018 and 2019. The genes associated with the risk for SCAD were the *F11R* (the gene responsible for F11 receptor which is a regulator of tight junction assembly), *TLN1* ( the gene encoding Talin 1 which is responsible for linking the actin cytoskeleton to the extracellular matrix), *TSR1* (the gene which influences ribosome maturation factor and RNA formation), already mentioned *PHACTR1* (the gene important for cytoskeleton actine), and *EDN1* (encoding endothelin 1 which is a circulating vasoactive peptide) (17).

Recently, Adlam et al. performed a meta-analysis of genome-wide association studies (GWAS) with approximately 2,000 patients with SCAD. The authors reported 17 risk genes and therefore polygenic inheritance for this disease. The genetics for atherosclerosis and SCAD showed some overlaps but in opposite directions. The genetic association was again confirmed for SCAD and FMD, but also for migraine and SCAD. Among the so-called new genes for SCAD, those associated with arterial hypertension gained more attention. It seems that there is a genetic link between arterial hypertension and the risk for developing SCAD. Strict control of arterial blood pressure could be pivotal in decreasing the risk of SCAD recurrence (42–44).

Published genetic data do not explain the female dominance demonstrated in this condition. This could be due to autosomal genes that have sex-related regulators (genes with estrogen-responsive structures) (17). Genetic testing for SCAD is not a standard practice, but it may be considered in situations where connective tissue diseases are suspected, as mentioned previously (18).

### 1.9. Atherosclerotic risk factors

Patients with SCAD have less percentage of the traditional cardiovascular risk factors for ischemic heart disease. New publications indicate that those patients could have traditional factors as well. In majority of them, hypertension, smoking, and dyslipidemia have been reported. However, no causal relationship has been found thus far (10, 18). According to a recently published study, SCAD patients exhibit arterial hypertension in approximately 37% of cases, while 35% have hyperlipidemia, which is almost identical as those observed in the population without SCAD (17, 45). Reasonably, those traditional risk factors for coronary artery disease are more common findings in older SCAD patients (10). It was reported that in a population involving over 600 SCAD patients, diabetes mellitus was identified in 0.9%–4.6% of cases, while smoking habits were observed in 0.6%–10% of patients. Consistent with earlier findings, hypertension was prevalent in 27%–36% of patients. Notably, obesity was not present in this population (46).

Arterial hypertension is highly prevalent in the general population. Also, high arterial systolic pressure and pulsatory pressure could cause SCAD by increasing the shear stress on the vessels. Notably, some psychosocial factors that are present predominantly in females could indirectly act by increasing the risk for hypertension, smoking, or abdominal obesity and therefore the risk for SCAD (46, 47).

In younger women, fat deposits primarily accumulates in the subcutaneous tissue, while in menopausal women, fat is starting to accumulate in visceral depots which is characteristic for men. In this way, the metabolic risk is increasing. Hormone replacement therapy with estrogen seems to recover the endothelial function in postmenopausal women (48). Visceral fat is an endocrine organ that releases adipokines and influences many metabolic pathways. Perivascular adipose tissue has effects on the development of atherosclerosis through inflammatory mechanisms, and it is also believed to play a significant role in SCAD development (49).

### 1.10. Mechanical stressors and exercise

The so-called mechanical stressors such as intense Valsalva-like maneuvers and factors that provoke coronary spasm have been reported in SCAD. In a recently published prospective studies with SCAD patients, the males in 11.9% of cases experienced chest pain immediately after isometric or intense physical activity (10). This physical stressor could induce a rise in catecholamine levels and a further increase in coronary artery shear stress, resulting to SCAD (18). Interestingly, the authors found retrograde SCAD on the coronary angiograms in Takotsubo syndrome associated with this type of stress (9). Sympathomimetic drugs such as amphetamines and cocaine have probably the same pathophysiology mechanisms in developing SCAD (15). The use of anabolic steroids, which could lead to the weakness of the arterial medial wall, in combination with intense physical activity (lifting weights) was reported in one case report as the cause of SCAD in a male patient (50). These substances enhance blood coagulability by reducing the production of plasminogen activator and influencing platelet function as well (51).

### 1.11. Emotional stressors

Preceding emotional stress has been described in a significant percentage of SCAD cases, particularly in women (10). Emotional distress, 24 h before ACS, was significantly more frequent in the SCAD group, but not in patients with type 1 MI. A high percentage of SCAD patients, 41%–55% in some published papers, have reported experiencing some kind of uncommon psychological stress prior to the occurrence of the event (3, 10).

It was reported that 40.5% of participants from a large prospective cohort study had some level of emotional stress followed by ACS, and 24% of patients were involved in physical activity, of whom 12.5% were engaged in isometric exercise. A smaller number of patients reported minor activities prior to SCAD such as severe coughing or vomiting. More recent studies reported that females with concomitant FMD and psychological stressors were at risk for SCAD. On the other part, men without FMD and physical stressors were at high risk for developing SCAD (13).

The results from a large Canadian study showed that men with SCAD were younger than females and that they more frequently had some physical but not psychological stressor preceding the ACS caused by SCAD (10). As mentioned, women with SCAD had a higher self-reported prevalence of anxiety and depression. Psychiatric disorders are known risk factors for bad prognosis and are linked with adverse cardiovascular events. There is a theory that women are more prone to psychological stressors which can lead to a rise in coronary artery shear stress and SCAD (12).

## 2. Discussion: clustering of SCAD risk factors

There were attempts to generate a risk model for developing SCAD. Smaardijk et al. (5) found that in included female patients with SCAD, conventional cardiovascular risk factors were less frequent (<10%), except for arterial hypertension (in 31% of patients). Half of the patients had high levels of self-reported psychological stress; they were complaining of fatigue and burnout syndrome. The frequency of psychological diseases, such as anxiety and depression, was relatively low (9% and 12%). The authors extrapolated three risk factor “clusters”: the first cluster was with FMD and non-conventional disorders such as tinnitus or chronic pain; the second “cluster” included migraine; and the third cluster was without any of these conditions.

Upon reviewing the existing literature, two distinct at-risk phenotypes for SCAD were identified: firstly, young women with antecedent psychological stressors, and secondly, middle-aged men with conventional risk factors, in whom the physical stressors preceded the event (8).

Giacalone et al. (46) recently divided the non-traditional risk factors in female patients as follows: “gender-specific,” such as pregnancy; “gender-predominant,” e.g., migraine; and “gender-related,” including those diseases and stressors that affect women more often, e.g., depression, psychosocial risk factors such as partner violence or low socioeconomic status. Depression is an important risk factor for cardiovascular morbidity and mortality; however, there are substantial inconsistencies and variability in reporting the prevalence of depression and anxiety in patients with SCAD and those with atherosclerotic MI (3, 52). Data from a large US national database, including more than 66,000 patients with SCAD, showed that anxiety and depression were less frequent in SCAD patients compared with patients with atherosclerotic MI (30). Recently, Muphy et al. (53) found that among SCAD patients, there was a higher prevalence of anxiety, depression, and other psychological disturbances compared with atherosclerotic MI. There is a need for further research in this area, utilizing standardized questionnaires completed early after hospital admission and incorporating subsequent psychiatric evaluations.

In Table 1, we summarized the most common predisposing conditions, traditional risk factors for coronary artery disease, and precipitating factors that are associated with or preceding chest pain in SCAD patients.

**Table 1:** The common predisposing conditions, traditional risk factors, and precipitating factors in SCAD patients.

Predisposing conditions	Available evidence
Fibromuscular dysplasia	Multiple cohort studies
Coronary tortuosity and ectasia	Single cohort study (tortuosity), case reports (ectasia)
Pregnancy (antepartum, postpartum, multiple pregnancies)	Multiple cohort studies
Connective tissue disorders Marfan's syndrome Loeys–Dietz syndrome Ehler Danlos syndrome type IV (vascular type) Polycystic kidney disease	Single case reports or small case series Multiple cohort studies and single genetic study confirm rare association between SCAD and heritable connective tissue disorders
Hormonal imbalance/therapy Oral contraception Estrogen replacement therapy Clomiphene *β*-HCG Testosterone Polycystic ovarian syndrome	Female sex predominance in multiple cohort studies Associations with exogenous hormones limited to case reports and reported prevalence of hormone therapy in cohort studies
Systemic diseases Systemic lupus erythematodes Inflammatory bowel disease Polyarteritis nodosa Sarcoidosis Churg–Strauss syndrome Granulomatosis with polyangiitis (Wegener) Rheumatoid arthritis Takayasu arteritis Hypothyroidism Celiac disease Cryoglobulinemia	Reported increased prevalence of inflammatory conditions in cohort studies Single case reports or small case series
Traditional risk factors	
Arterial hypertension Hyperlipemia Depression (30) (ref)	Multiple cohort studies
Precipitating factors	
Intense exercise (isometric, aerobic)	Multiple cohort studies, single case reports, and small case series
Emotional stress	Multiple cohort studies
Coronary Spasm	Small case series
Recreational drugs Cocaine Amphetamines	Isolated cases in cohort studies and case reports
Valsalva type activities (sexual activity, vomiting, cough etc.)	Single case reports
Drugs: calcineurin inhibitors, 5-FU, fenfluramine, corticosteroids, methylphenidate, ergotamine, sumatriptan, dobutamine	Single case reports
Sleep deprivation	Single case report
Hypersensitivity reactions	Single case report

β-HCG, *β*-subunit of hCG gonadotropin; 5-FU, 5-fluorouracil.

Table was modified from Supplementary material online for: ESC-ACCA position statement on spontaneous coronary artery dissection: Eur Heart J 2018;39(36):3353–3368.

SCAD incidence increased 10-fold in the last two decades. This could be attributed to a significant improvement in the diagnostic capabilities and increased awareness of this disease among healthcare providers. However, this disease is poorly studied and understood. In 2018, the American Heart Association (AHA) and the European Society of Cardiology (ESC) released expert consensus statements regarding SCAD. Subsequently, in 2019, Canadian authors published findings from a large multicenter, prospective registry focused on SCAD patients (54). These initiatives contribute significantly to and improve the awareness among healthcare providers regarding the diagnosis and management of SCAD patients.

Specifically, it all started with improved diagnostic techniques and defining the myocardial infarction with non-obstructive coronary arteries (MINOCA). It accounts for approximately 15% of all acute myocardial infarctions (AMI) and more frequently affects females. The pathogenesis of MINOCA is heterogeneous and may include atherosclerotic plaque rupture, plaque erosion with thrombosis, vasospasm, embolization, SCAD, or a combination of mechanisms. Non-vascular causes include acute myocarditis, Takotsubo syndrome, and non-ischemic cardiomyopathies which can mimic the clinical presentation. In one study, 67% of patients with initial MINOCA were reclassified after the cardiac magnetic resonance (CMR) evaluation. Combining optical coherence tomography (OCT) with CMR was shown to identify the majority of the underlying mechanisms of MINOCA. Furthermore, CMR-confirmed diagnosis of MINOCA was associated with an increased risk of major adverse cardiovascular events at follow-up (55–58).

Computed tomography coronary angiography (CTCA) for the diagnosis of acute SCAD has the advantage of being non-invasive, without the increased risk of iatrogenic dissection. However, its role in acute setting is limited due to its reduced spatial resolution. Currently, it could play a role in the follow-up of SCAD patients (10, 59).

## 3. Conclusion

According to a recently conducted population-based analysis using the National Inpatient Sample (NIS), in-hospital mortality was higher in SCAD patients compared with patients with MI caused by atherosclerosis of coronary arteries (60). The logistic regression analysis identified atrial fibrillation, steroid use, ventricular arrhythmias, and cardiac arrest as significant predictors of intra-hospital mortality (8).

It is crucial to identify emotional and physical stressors that can trigger this not-so-rare disease, particularly in those with potential predisposing conditions such as FMD (21).

The classic angiographic characteristics of SCAD with defined intimal tear or spiraling dissection may not be evident, and intravascular ultrasound (IVUS) and optical coherence tomography should be used with extreme caution when SCAD is suspected (10, 59). The utilization of new imaging modalities and careful examination of patients with chest pain led to a better understanding of the SCAD pathophysiology and improved its diagnosis. However, there is a lack of randomized controlled studies for the treatment of SCAD, and further investigation is needed. Percutaneous coronary intervention is recommended only when patients have symptoms and signs of ongoing myocardial ischemia, or a large area of myocardium in jeopardy, and reduced antegrade flow (10).

SCAD has not been heavily linked to traditional risk factors, but it exhibits a strong association with specific comorbidities such as migraine and tinnitus, particularly in younger females who experience emotional triggers within 24 h preceding the ACS. This could be a profile that should be recognized by cardiologists leading to prompt diagnosis and appropriate treatment. These differences in profiling SCAD compared with atherosclerotic MI patients may decrease the occurrence of misdiagnosis and underdiagnosis of SCAD, hence improving its prognosis. Cardiac rehabilitation should be personalized for SCAD patients, and these patients should be educated regarding the known risk factors (3).
